# Organic fertilizers and *Azotobacter*: effects on onion growth, yield, metabolites, and soil fertility

**DOI:** 10.1186/s13568-025-01895-5

**Published:** 2025-05-30

**Authors:** Waleed M. Ali, Sadoun M. E. Sultan, Ahmed M. Ali, Hassan M. Al-Sayed, Mohamed Ahmed Mahmoud, Hanan G. Ismail, Islam I. Teiba, Ahmed Fathy Yousef

**Affiliations:** 1 Department of Horticulture, College of Agriculture, University of Al-Azhar (Assiut Branch), Assiut, 71524 Egypt; 2 Department of Soils and Water Sciences, Faculty of Agriculture, Al-Azhar University (Assiut Branch), Assiut, 71524 Egypt; 3 Agronomy Department of Agronomy (Biochemistry Branch) Faculty of Agriculture, Al-Azhar University, (Assiut Branch), Assiut, Egypt; 4 Department of Soil and Agricultural Chemistry, Faculty of Agriculture (Saba Basha), Alexandria University, Alexandria, 21531 Egypt; 5 Microbiology, Botany Department, Faculty of Agriculture, Tanta University, Tanta, 31527 Egypt

**Keywords:** Rabbit manure, Sustainable agriculture, Vermicompost, Nitrogen fixation, Cation exchange capacity, Agricultural microorganisms

## Abstract

This study assesses the effects of organic fertilizers (vermicompost (V) and rabbit manure (R), combined with the bio-fertilizer *Azotobacter chroococcum* (AZ), on onion growth, yield, soil properties, and chemical metabolites over two growing seasons (2021/2022 and 2022/2023). The experiment, conducted at the Experimental Farm of Al-Azhar University, Assiut, Egypt, used a split-plot design with organic fertilizers as the main plot and bio-fertilizer treatments as subplots. The combined application of rabbit manure and Azotobacter (R + AZ) produced the tallest plants (59.64 cm and 63.23 cm), highest leaf number (10.71 and 10.82), and greatest bulb diameter (10.29 cm and 10.39 cm) in the first and second seasons, respectively. This treatment also resulted in the highest bulb length (9.49 cm and 9.66 cm), bulb weight (2.54 kg and 2.61 kg per 10 plants), and total yield (63.86 and 65.64 ton ha^−1^) in the first and second seasons, respectively. Additionally, organic fertilization improved several soil properties, including increased organic matter content, pH reduction, and changes in electrical conductivity. *Azotobacter* inoculation also increased the concentration of bioactive metabolites, such as phenolic compounds, flavonoids, and lipids, with the R + AZ treatment showing the most significant increases. This study demonstrates that the combination of rabbit manure and *Azotobacter* can enhance onion yield and nutritional quality, providing a sustainable approach to onion production by improving soil health and reducing the need for synthetic fertilizers.

**Graphical abstract:**

A graphic abstract explains the importance of adding organic fertilizersand *Azotobacter* to improve the growth and yield of onion plants, as wellas the fertility and health of the soil.

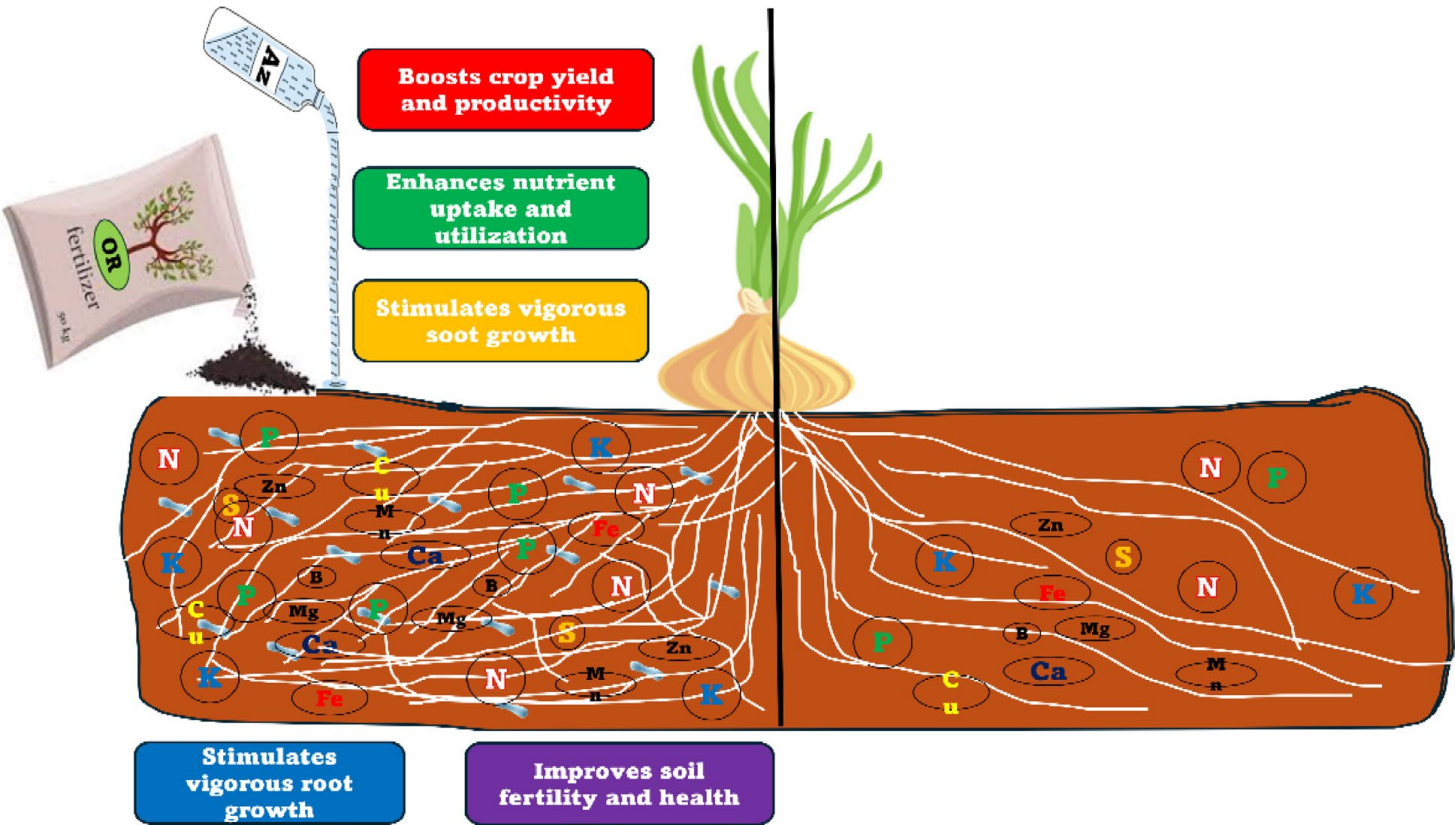

## Introduction

Onions (*Allium cepa* L.) are a major vegetable crop widely cultivated in arid and semi-arid regions around the world (Ochar and Kim 2023; Gideon et al. 2023). Global production has expanded significantly, from under 2 million hectares in 1990 to more than 5 million hectares by 2022 (Dhotre et al. 2025). Today, onions hold the second-highest gross production value among all vegetable crops (Dhotre et al. 2025). This crop holds significant importance on both local and global scales. Their importance spans both local and international markets, owing not only to their economic value but also to their nutritional and medicinal properties. Onions contain essential vitamins (A, E, K), minerals, and beneficial fats, and possess thrombolytic, hypocholesterolemic, and antioxidant effects, which contribute to cancer prevention and cardiovascular health (Srinivasan 2024).

In Egypt, onion cultivation plays a significant role in agricultural production and export. In 2022, the harvested area for dry onions reached approximately 87,948 hectares, yielding about 3,081,047 tons (FAO 2021). By 2023, exports of fresh or chilled onions and leeks totaled around 206,843 tons, valued at $242.4 million (United 2023). This marks a notable increase from 2022, when Egypt ranked seventh among global onion exporters with export values reaching $281 million (OEC 2022).

Their combined use has been shown to improve plant growth, nutrient uptake, and overall crop productivity (Mukungu 2024). To achieve high onion yields, nitrogen-rich fertilizers are often required. However, heavy reliance on chemical fertilizers has led to environmental issues and soil fertility degradation, threatening long-term agricultural sustainability (Pahalvi et al. 2021). As a result, growers are increasingly adopting organic alternatives to mitigate these effects (Zaib et al. 2023). Organic fertilizers such as vermicompost and rabbit manure are gaining attention due to their ability to enhance soil structure, nutrient content, and microbial activity. Vermicompost, in particular, is rich in macro- and micronutrients and improves soil organic matter, while both it and rabbit manure contribute to reduced nitrogen losses and enhanced soil properties (Abad and Shafiqi 2024; Walia and Kaur 2024; Rekaby et al. 2024; Alejo Jr and Nicolas 2021). The synergistic use of organic fertilizers, including vermicompost, has been shown to improve plant growth, nutrient uptake, soil fertility, and overall yield (Mukungu 2024).

Despite the known benefits, studies focusing on organic fertilizer use in onion cultivation, especially in arid regions like Egypt, remain limited (Díaz-Pérez et al. 2021; Ali et al. 2023). Despite extensive research on organic farming practices and their effects on crops and soil, the specific impacts of rabbit dung on onion production in Egypt remain largely unexplored. In this context, the utilization of bio-fertilizers is considered eco-friendly and aims to enhance plant productivity while maintaining ecological balance and ensuring food safety for humans (Yousef et al. 2023, 2020). Bio-fertilizers, which include microbial inoculants, composed of bacteria, assist in the biological fixation of atmospheric nitrogen (N₂) and the mobilization of other essential nutrients (Gulaiya et al. 2024; Rohela and Saini 2022). Nitrogen-fixing organisms, such as *Azotobacter*, are particularly significant in vegetable crop production (Mukherjee et al. 2023).

Free-living nitrogen-fixing bacteria like *Azotobacter* not only fix nitrogen but also produce phytohormones, such as gibberellins, indole acetic acid, and cytokinins, which stimulate plant growth and enhance nutrient availability for root uptake through improved nutrient solubility and increased photosynthesis (Jehani et al. 2023; Aasfar et al. 2021). The application of bio-fertilizers containing diverse microbial strains has been associated with a decrease in chemical fertilizer usage and the production of high-quality agricultural products free from harmful agrochemicals, thereby ensuring safety for human consumption (Daniel et al. 2022). Studies indicate that the integration of inorganic fertilizers with bio-fertilizers significantly influences plant growth, soil fertility, and crop productivity (Yousef et al. 2020; Imran 2024; Sharma et al. 2023). However, limited information exists regarding the combined effects of vermicompost and rabbit manure on onion plants.

This study hypothesizes that partially substituting chemical fertilizers with vermicompost or rabbit manure will enhance the growth of onions inoculated with bio-fertilizers. Therefore, this research aims to evaluate the effects of combining various organic materials with bio-fertilizers on the growth, bulb yield, quality, and mineral nutrient content of Egyptian onion plants.

## Materials and methods

### Experimental site

**Fig. 1:**
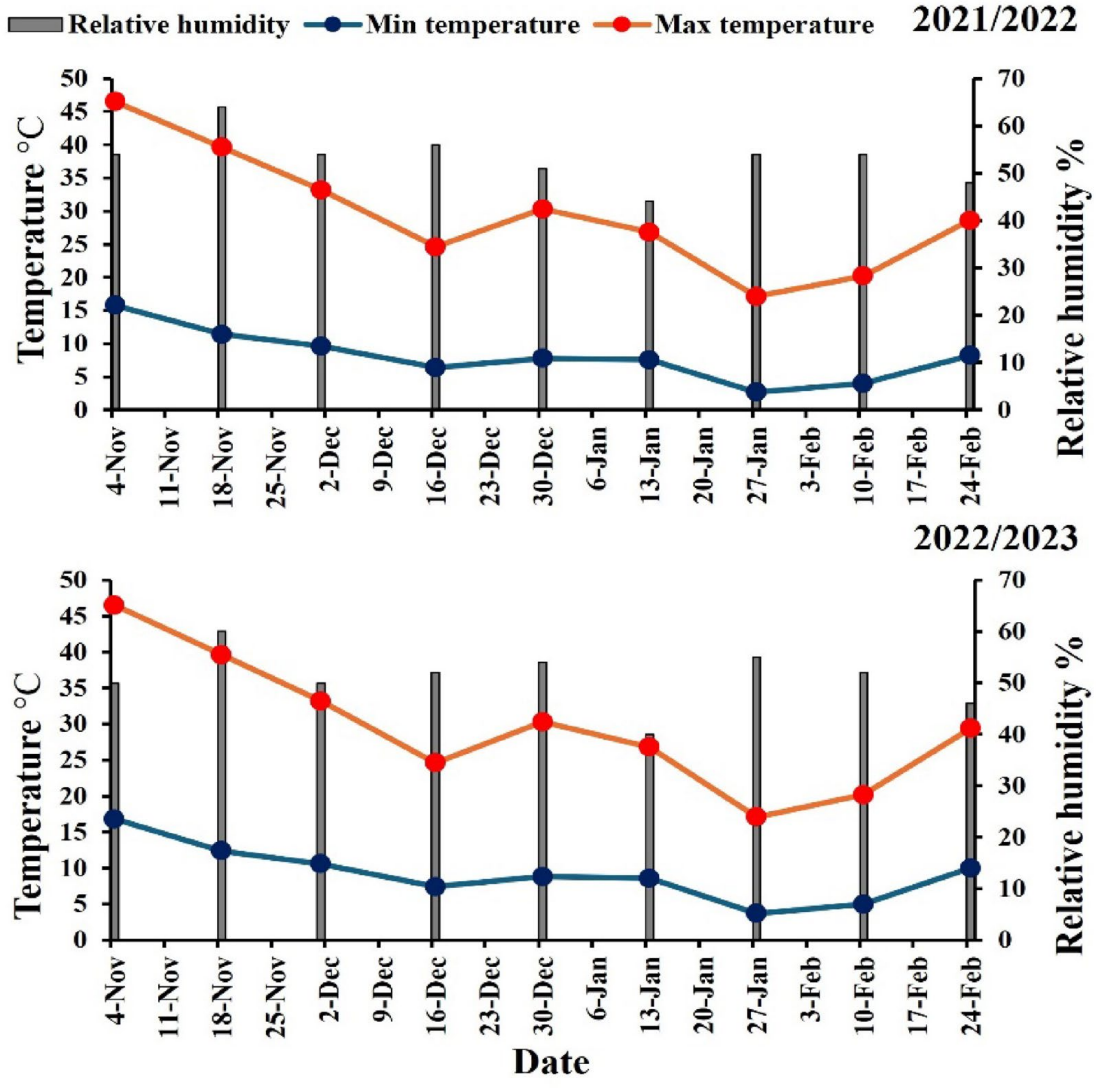
The weather of two growing seasons 2021/2022 and 2022/2023

The field experiment was conducted over two growing seasons at the Experimental Farm of the Faculty of Agriculture, Al-Azhar University, Assiut, Egypt, located at 27°12′16.67″N latitude and 31°09′36.86″E longitude. Based on the Köppen-Geiger climate map, the climate of the experimental area is categorized as extremely hot and dry in the summer, with cold temperatures during the winter months (Beck et al. 2018). The weather of two growing seasons (2021/2022 and 2022/2023) is shown in Fig. 1.

A split-plot design with three replications was used to set up the field studies. Onion seeds (*Allium cepa* L.; Giza 20) were obtained from the Agricultural Research Center, Giza, Egypt. The seeds were immersed in 2% sodium hypochlorite solution for 10 min, washed with sterile distilled water, and then planted in a nursery greenhouse in sterile sandy soil for seedling development. After 60 days of sowing, seedlings were transplanted in an open field on November 15th, 2021, and 2022, respectively. The experimental plot measured 3.5 × 4.5 m with 4 rows at a distance of 50 cm apart, had ridge spacing of 7 cm in a row, and was planted on two feathers (416 plants plot^−1^).

The bio-fertilizer was nitrogen-fixing Gram-negative bacterium [*Azotobacter chroococcum* (ATCC^®^ 9043™)] obtained from the cultural collocation of Agric. Microbiology Dep. National Research Center, Giza, Egypt; in the form of a liquid broth culture containing 1 × 10^7^ CFU/mL, which was purchased from (https://www.atcc.org/products/9043).

The experimental design was a split-plot design with three replicates. The main plot was allocated by the soil application of organic fertilizers: Control treatment: mineral fertilizer at the full recommended rate (C); 100% vermicompost; 10.20 ton ha^−1^(V); 100% rabbit manure; 7.21 ton ha^−1^ (R); 50% vermicompost + 50% rabbit manure (VR). While subplot was allocated by the soil application of bio-fertilizer nitrogen-fixing Gram-negative bacterium [*Azotobacter chroococcum* (without Non-AZ and with AZ)].

According to the Egyptian Ministry of Agriculture and Land Reclamation, 143 kg N ha^−1^ from either vermicompost (1.40% N) or rabbit manure (1.98% N) were applied to each plot alone and/or both sources as organic sources were added during soil preparation (Hassan 2011). Urea fertilizer (46% N) as a chemical source was split into three uniform doses which were applied at 30, 60, and 90 days after sowing (DAS) (143 kg N ha^−1^), whereas was applied to each plot 120 kg P_2_O_5_ kg ha^−1^ from super-phosphate (15.5% P_2_O_5_) during soil preparation. A total of 143 kg (K ha^−1^) in the form of potassium sulfate was divided into two equal doses; the 1st dose was added through soil preparation and the 2nd dose was added at 90 DAS.

### Soil analysis

Composite soil samples were collected from a depth of 0–30 cm, air-dried, crushed, and sieved through a 2 mm mesh (Carter and Gregorich 2007). Soil texture was analyzed using the pipette method as described by Page (1982). Soil organic matter (OM) content was determined using the Walkley–Black dichromate oxidation method, where soil samples were oxidized with a known excess of potassium dichromate (K_2_Cr_2_O_7_) and concentrated sulfuric acid (H_2_SO_4_). The remaining dichromate was then titrated with ferrous sulfate to estimate the organic carbon content, which was multiplied by a conversion factor to estimate OM (Jackson 1973). Calcium carbonate (CaCO_3_) content was measured using the calcimeter method described by Jackson (1973), in which carbon dioxide (CO_2_) released upon acidification of soil samples with hydrochloric acid (HCl) was quantified volumetrically using a calcimeter device. Soil salinity was assessed by measuring electrical conductivity (EC) of a 1:1 (w/v) soil-to-distilled water extract using an EC meter (LOvibond 200 Con, Germany), in accordance with standard procedures. Soil pH was measured in a 1:2 (w/v) soil-to-water suspension using a calibrated digital pH meter (Hanna Instruments pH 211, Romania), following the method outlined by (Jackson 1973). Available nitrogen (N) was extracted using 1% potassium sulfate (K₂SO₄) solution at a 1:5 soil-to-extractant ratio. A 20 mL aliquot of the filtrate was distilled in the presence of 1 g of Devarda’s alloy using a micro-Kjeldahl apparatus, which reduces nitrate and nitrite to ammonium for quantification (Jackson 1973). Available phosphorus (P) was extracted with 0.5 M sodium bicarbonate (NaHCO₃) at pH 8.5, and quantified colorimetrically using the stannous chloride (SnCl_2_) method. The blue color intensity was measured at a wavelength of 660 nm using a UV–visible spectrophotometer (Unico 2000UV, Germany) (Jackson 1973). Available potassium (K) was determined by flame photometry using a Jenway 7PFP flame photometer (England). The soil extract was prepared using ammonium acetate (1 N) as the extracting solution, following the procedure described by Jackson (1973). The key physical and chemical properties of the investigated soils are presented in Table 1, while the chemical characteristics of the organic fertilizers used in the study are shown in Table 2.

**Table 1:** The relevant physical and chemical properties of the investigated soils

Properties	Units	Value
Sand	g kg^−1^	254
Silt	g kg^−1^	392
Clay	g kg^−1^	354
Soil texture	Clay loam	
CaCO_3_	g kg^−1^	38.00
pH (1:2.5)		8.23
EC (1:1)	dS m^−1^	1.27
Organic matter	g kg^−1^	11.80
Available N	mg kg^−1^	29.50
Available P (Olsen)	mg kg^−1^	5.70
Available K	mg kg^−1^	166.50

Each value represents a mean of three replicates

**Table 2:** Some chemical properties of tested organic materials

Properties	Vermicompost	Rabbit manure
pH (1:2.5)	8.16	8.05
EC(1:5) (dS m^−1^)	6.81	8.79
OM (g kg^−1^)	345.00	454.50
Total N (g kg^−1^)	14.00	19.80
Total P (g kg^−1^)	12.05	15.20
Total K (g kg^−1^)	5.50	10.20

### Plant analysis

After 120 days of transplanting, ten plants were randomly selected from each plot to evaluate the vegetative growth and yield properties of the onion plants. The following parameters were measured: plant height (cm), bulb diameter (cm), bulb length (cm), neck diameter (cm), number of leaves per plant, average bulb weight (kg), and total bulb yield per hectare, calculated based on the plot's yield. At harvest, composite plant samples, consisting of ten plants, were collected from each experimental unit. The samples were cleaned and washed with tap and distilled water, air-dried, and then oven-dried at 70 °C before being ground. For nutrient analysis, precisely 0.2 g of the dried and ground bulb material was digested using 10 mL of a concentrated sulfuric acid and perchloric acid mixture (7:3 v/v) following the method of Jackson (1973). The digestion was carried out under a fume hood using a block digestion system until the solution turned clear. The digestate was diluted to 50 mL with distilled water and filtered for analysis. Total nitrogen (N) was determined using the micro-Kjeldahl method, involving distillation with 40% sodium hydroxide, and titration of the released ammonia trapped in boric acid solution. Total phosphorus (P) was quantified colorimetrically using the vanadomolybdophosphoric acid yellow method, with absorbance read at 420 nm using a UV–Visible spectrophotometer (Unico 2000UV, Germany). Total potassium (K) was measured using a flame photometer (Jenway 7PFP, England) after appropriate dilution of the digest. All measurements were conducted in triplicate, and results were expressed on a dry weight basis.

### Physiological analysis

The chemical composition of onion was analyzed following the official methods of the Association of Official Analytical Chemists (AOAC) as follows:

#### Determination of total ash content (TAC)

Total ash content was determined following the AOAC (1984) method. Approximately 1.0–2.0 g of the sample was weighed into a pre-cleaned, pre-weighed porcelain crucible and placed in a muffle furnace set at 600 ± 10 °C. The sample was heated for 3 h or until complete ashing, indicated by the absence of visible black residues. After ashing, the crucible was removed, cooled in a desiccator to room temperature, and reweighed to determine the residue. TAC was calculated as a percentage of the original sample weight. The following equation is used: TAC (%) = [Weight of Ash (g)/Weight of Sample (g)] × 100.

#### Determination of crude lipids (CL)

The crude lipid content was determined using the method outlined by the Association of Official Analytical Chemists (AOAC 1984). Briefly, dried samples weighing between 1 and 2 g were accurately measured and subjected to lipid extraction using a Soxhlet apparatus. Petroleum ether with a boiling point range of 60–80 °C was employed as the solvent, and the extraction process was carried out continuously for 15 h to ensure complete lipid recovery. Following extraction, the solvent was evaporated under reduced pressure using a rotary evaporator to isolate the lipids. The remaining crude lipid content was then weighed to determine the total lipid concentration in the sample. This method ensures precise and reproducible quantification of lipids in the analyzed samples. The following equation is used: Crude Lipid (%) = [Weight of Extracted Lipids (g)/Weight of Dry Sample (g)] × 100.

#### Extraction and determination of total carbohydrates

The total carbohydrate content was determined using acid hydrolysis followed by the phenol–sulfuric acid method as described by DuBois et al. (1956). Briefly, 1.0 g of dried sample was hydrolyzed with 10 mL of 1N H_2_SO_4_ in a water bath at 100 °C for 30 min. After cooling, the solution was neutralized with 0.1 g of BaCO_3_, filtered through Whatman No. 1 filter paper, and diluted to 100 mL with distilled water. The total carbohydrate content was quantified using the phenol–sulfuric acid method, where an aliquot of the sample was mixed with 5% phenol and concentrated H_2_SO_4_, and the absorbance was measured at 490 nm. A standard curve was prepared using glucose solutions (10–100 µg/mL) for quantification, and the results were expressed as a percentage of the dry sample weight. The following equation is used: Total Carbohydrates (%) = [Concentration of Carbohydrates from Standard Curve (µg/mL) × Dilution Factor/Weight of Sample (g)] × 100.

#### Extraction and determination of total phenolic compounds (TPCs)

Total phenolic compounds (TPCs) were extracted from a 1.0 g onion sample by refluxing with 30 mL of methanol containing 1% HCl for 10 min. The resulting extract was then centrifuged at 8000 rpm for 10 min. The concentrations of TPCs in the methanolic extracts were determined following the method outlined by Singleton and Rossi (1965), with minor modifications. One milliliter of the sample was mixed with 1 mL of Folin-Ciocalteu’s phenol reagent. After 3 min, 1 mL of saturated Na₂CO₃ solution (35%) was added, and the mixture was diluted to 10 mL with distilled water. The reaction was kept in the dark for 90 min, after which the absorbance was measured at 725 nm. A calibration curve was constructed using different concentrations of gallic acid (0.01–1 mM) as the standard. The following equation is used: Total Phenolic Compounds (mg GAE/g) = [Concentration of TPCs from Standard Curve (mg/mL) × Volume of Extract (mL)/Weight of Sample (g)].

#### Extraction and determination of total flavonoids (TFs)

Oven-dried seed samples (30 g) were extracted using a Soxhlet apparatus with 100 mL of distilled ethanol for 1 h, and the resulting extract was filtered. A known volume of the extract was transferred to 10 mL volumetric flasks. Distilled water was added to make up 5 mL, followed by the addition of 0.3 mL NaNO_2_ solution (1:20). After 5 min, 3 mL of AlCl_3_ solution (1:10) was added. Six minutes later, 2 mL of 1 M NaOH was introduced, and the volume was adjusted to 10 mL with distilled water. The solution was thoroughly mixed, and the absorbance was measured against a blank at 510 nm using a spectrophotometer. Flavonoid content was expressed as mg of quercetin equivalents (QE) per gram of dried seeds (Zhishen et al. 1999). The following equation is used: Total Flavonoids (mg QE/g) = [Concentration of TFs from Standard Curve (mg/mL) × Volume of Extract (mL)/Weight of Sample (g)].

### Statistical analysis

All data were statistically analyzed using the variance test [Two-way ANOVA(Organic fertilizers and Biofertilizer)], which was carried out using the package (Statistix 8.1) according to Gomez and Gomez (1984). Differences between individual means were separated by Duncan's New Multiple Range Test with a probability of *p* < *0.05* (Steel and Torrie 1980).

## Results

### Impact of treatments on morphological parameters

The results in Table 3 indicate a significant impact of the treatments on the height of onion plants across both seasons. In 2021/2022, the C exhibited a mean plant height of 44.98 cm, while the application of R led to the highest average height of 56.07 cm, demonstrating the positive effect of this organic amendment. The V resulted in an average height of 49.95 cm, and the combined treatment of RV showed an intermediate mean of 51.70 cm. The application of Non-AZ exhibited a mean plant height of 47.37 cm, while the application of AZ led to the highest average height of 53.98 cm. Similarly, in 2022/2023, the control group recorded a mean height of 45.37 cm, while rabbit manure again yielded the greatest height at 59.66 cm. The vermicompost treatment recorded a mean height of 51.67 cm, and the RV treatment was slightly lower at 52.04 cm. Overall, the data indicate that the use of rabbit manure significantly enhances the plant height of onions, outperforming both the control and other treatments. The application of exhibited a mean plant height of 49.28 cm, while the application of AZ led to the highest average height of 55.09 cm. The results of the interaction between the two factors showed that the lowest values were under the control application without organic fertilizer (C + Non-AZ) during the two seasons (42.15 and 43.66 cm, respectively), while the highest values were under the application of rabbit manure with bio-fertilizer (R + AZ) during the two seasons (59.64 and 63.23 cm, respectively).

**Table 3:** The integrated effect of Rabbit manure, Vermicompost treatments and their combinations with and without biofertilizer on growth variables of onion plants after two successive seasons (2021/2022–2022/2023)

Season	Treatment	Plant height		Mean (A)	Number of leaves		Mean (A)	Bulb diameter		Mean (A)
		Non-AZ	AZ		Non-AZ	AZ		Non-AZ	AZ	
2021/2022	C	**42.15 ± 1.52e**	47.82 ± 1.42d	44.98d	7.97 ± 0.16ef	8.32 ± 0.34de	**8.14c**	**8.99 ± 0.44c**	9.71 ± 0.36abc	**9.35a**
	R	52.49 ± 1.26c	**59.64 ± 1.53a**	**56.07a**	8.93 ± 0.28c	**10.71 ± 0.48a**	**9.82a**	9.30 ± 0.45bc	**10.29 ± 0.39a**	**9.79a**
	V	46.76 ± 1.67d	53.13 ± 1.14c	49.95c	**7.67 ± 0.32f**	8.00 ± 0.25ef	**7.84c**	9.22 ± 0.58bc	10.24 ± 0.73a	**9.73a**
	RV	48.09 ± 1.19d	55.31 ± 1.20b	51.70b	8.68 ± 0.35cd	9.65 ± 0.47b	**9.16b**	9.0 ± 0.40bc	9.92 ± 0.45ab	**9.51a**
	Mean (B)	**47.37b**	**53.98 a**		**8.31b**	**9.17a**		**9.15b**	**10.04a**	
	LSD (*p* ≤ 0.05)	**A = 1.47, B = 1.04, AB = 2.08**			**A = 0.41, B = 0.29, AB = 0.58**			**A = 0.62, B = 0.44, AB = 0.88**		
2022/2023	C	**43.66 ± 2.29d**	47.08 ± 2.02C	45.37c	**8.24 ± 0.38d**	8.72 ± 0.31cd	**8.48c**	**8.92 ± 0.40d**	9.61 ± 0.45bc	**9.27a**
	R	56.09 ± 1.17b	**63.23 ± 1.12a**	**59.66a**	9.13 ± 0.16c	**10.82 ± 0.24a**	**9.97a**	9.08 ± 0.44cd	**10.39 ± 0.36a**	**9.73a**
	V	48.56 ± 1.67c	54.77 ± 2.12d	51.67b	8.32 ± 0.28d	9.02 ± 0.18c	**8.67c**	9.47 ± 0.58bcd	9.96 ± 0.73ab	**9.71a**
	RV	48.81 ± 0.94c	55.27 ± 2.30b	52.04b	8.96 ± 0.28c	9.91 ± 0.36b	**9.44b**	9.43 ± 0.45bcd	9.90 ± 0.39ab	**9.66a**
	Mean (B)	**49.28b**	**55.09a**		**8.66b**	**9.62a**		**9.22b**	**9.96a**	
	LSD (*p* ≤ 0.05)	**A = 2.21, B = 1.56, AB = 3.13**			**A = 0.36, B = 0.25, AB = 0.51**			**A = 0.48, B = 0.34, AB = 0.69**		

The values shown in the table are the means of three replicates. Means followed by the same letters are non-significantly different (*p* ≤ *0.05*). Where: C = (100% mineral fertilizer at the full recommended rate); R = (100% Rabbit Manure); V = (100% Vermicompost); RV = (50% Rabbit Manure + 50% Vermicompost); Non-AZ = without *Azotobacter chroococcum;* AZ = with *Azotobacter chroococcum*

The results in Table 3 show that vermicompost and rabbit manure significantly affected the number of leaves across both growing seasons. In 2021/2022, the C exhibited a mean number of leaves of 8.14, while the application of Rled to the highest number of leaves, 9.82. This increase was highly significant (*p* ≤ *0.05*). The application of Non-AZ exhibited a mean number of leaves of 8.31, while the application of AZ led to the highest average of 9.17. Similarly, in 2022/2023, the C exhibited a mean number of leaves of 8.48, while the application of R led to the highest number of leaves, 9.97. These findings consistently highlight the positive effect of treatment R on leaf growth across both seasons (*p* ≤ *0.05*). The increase in nutrient availability supported the growth of the number of leaves, along with the diameter and weight of onion bulbs. The application of Non-AZ exhibited a mean number of leaves of 8.66, while the application of AZ led to the highest average of 9.62. The results of the interaction between the two factors showed that the lowest values were under the V application without organic fertilizer (V + Non-AZ) with 7.67 leaves, while the highest values were under the application of rabbit manure with bio-fertilizer (R + AZ) with 10.71 leaves during the first season. In the second season, the highest values were under the application of rabbit manure with bio-fertilizer (R + AZ) during both seasons (8.24 and 10.82), respectively.

The results presented in Table 3 demonstrate that both vermicompost and rabbit manure significantly influenced bulb diameter. In 2021/2022, the C exhibited a mean bulb diameter of 9.35 cm, while the application of R led to the highest bulb diameter at 9.79 cm, without a significant difference compared to other treatments. Similar, but less pronounced effects were observed in the V and RV treatments. The application of Non-AZ resulted in a mean bulb diameter of 9.15 cm, while the application of AZ led to the highest bulb diameter of 10.04 cm. In 2022/2023, the R treatment again resulted in the largest bulbs, with a mean diameter of 9.73 cm, while the control group exhibited the smallest diameter at 9.27 cm. Overall, the R treatment had the most substantial positive impact on bulb diameter, followed by RV and V, with the control showing the least effect. The application of Non-AZ exhibited a mean bulb diameter of 9.22 cm, while the application of AZ led to the highest average diameter of 9.96 cm. The results of the interaction between the two factors showed that the lowest values were under the control application without organic fertilizer (C + Non-AZ) during the two seasons (8.99 cm and 8.92 cm), respectively, while the highest values were under the application of rabbit manure with bio-fertilizer (R + AZ) during the two seasons (10.29 cm and 10.39 cm), respectively.

Data presented in Table 4 illustrate the significant impact of vermicompost and rabbit manure on bulb length across both growing seasons. In 2021/2022, the C exhibited a mean bulb length of 7.69 cm, while the application of R led to the highest bulb length at 8.89 cm. The application of Non-AZ exhibited a mean bulb length of 7.70 cm, while the application of AZ (with bio-fertilizer) led to the highest bulb length of 8.70 cm. In 2022/2023, the C exhibited a mean bulb length of 7.86 cm, while the application of R again led to the highest bulb length at 9.02 cm. The application of Non-AZ exhibited a mean bulb length of 7.92 cm, while the application of AZ led to the highest bulb length of 8.59 cm. The results of the interaction between the two factors showed that the lowest values were under the control application without organic fertilizer (C + Non-AZ) during both seasons (7.02 cm and 7.63 cm, respectively), while the highest values were under the application of rabbit manure with bio-fertilizer (R + AZ) during both seasons (9.49 cm and 9.66 cm, respectively).

**Table 4:** The integrated effect of Rabbit manure, Vermicompost treatments and their combinations with and without biofertilizer on growth variables of onion plants after two successive seasons (2021/2022–2022/2023)

Season	Treatment	Bulb length		Mean (A)	Nick diameter		Mean (A)
		Non-AZ	AZ		Non-AZ	AZ	
2021/2022	C	**7.02 ± 0.41e**	8.24 ± 0.32c	7.69c	2.64 ± 0.15cd	3.09 ± 0.17ab	2.87b
	R	8.28 ± 0.40c	9.49 ± 0.35a	8.89a	2.79 ± 0.26bcd	3.02 ± 0.33abc	2.90b
	V	8.02 ± 0.17 cd	8.19 ± 0.32c	8.11b	3.40 ± 0.49a	3.15 ± 0.30ab	3.28a
	RV	7.48 ± 0.37de	8.89 ± 0.27b	8.19b	**2.53 ± 0.17d**	2.87 ± 0.30bcd	2.70a
	Mean (B)	7.70b	8.70a		2.84a	3.03a	
LSD (*p* ≤ 0.05)		**A = 0.41, B = 0.29, AB = 0.58**			**A = 0.30, B = 0.21, AB = 0.42**		
2022/2023	C	7**.63 ± 0.21d**	8.03 ± 0.22cd	7.86c	2.65 ± 0.28ab	2.51 ± 0.34ab	2.58a
	R	8.38 ± 0.27bc	9.66 ± 0.30a	9.02a	2.84 ± 0.37ab	2.78 ± 0.21ab	2.81a
	V	7.96 ± 0.19cd	8.00 ± 0.21cd	7.98bc	3.03 ± 0.20a	2.79 ± 0.41ab	2.91a
	RV	7.70 ± 0.23d	8.69 ± 0.41b	8.16b	2.89 ± 0.21ab	**2.41 ± 0.36b**	2.65a
	Mean (B)	7.92b	8.59a		2.62a	2.50a	
LSD (*p* ≤ 0.05)		**A = 0.30, B = 0.21, AB = 0.42**			**A = 0.37, B = 0.26, AB = 0.52**		

The values shown in the table are the means of three replicates. Means followed by the same letters are non-significantly different (*p* ≤ *0.05*). Where: C = (100% mineral fertilizer at the full recommended rate); R = (100% Rabbit Manure); V = (100% Vermicompost); RV = (50% Rabbit Manure + 50% Vermicompost); Non-AZ = without *Azotobacter chroococcum;* AZ = with *Azotobacter chroococcum*

Table 4 illustrates that the effects of treatments on nick diameter varied significantly among the groups. In 2021/2022, there were significant differences in the first season for the organic fertilization treatments, but no significant differences were observed in the second season. The highest onion neck diameter in both seasons was observed under the application of V, with values of 3.28 cm in the first season and 2.91 cm in the second season. There were no significant differences between the biofertilization treatments in both seasons, but the lowest values were observed under the application of Non-AZ, and the highest values were observed under the application of AZ, which were 2.84 cm and 2.62 cm in the first season, and 3.03 cm and 2.50 cm in the second season, respectively. When analyzing the interaction between organic fertilization and biofertilization, the highest values for onion neck diameter were observed under the application of V in both seasons, with 3.40 cm in the first season and 3.03 cm in the second season. The lowest values for onion neck diameter were found in the first season under the application of RV + Non-AZ (2.53 cm) and in the second season under the application of RV + AZ (2.41 cm).

### Impact of treatments on yield onion plants

The results presented in Table 5 indicate that the weight of 10 plants was significantly affected by the various treatments across both growing seasons. In 2021/2022, the C exhibited a mean weight of 10 plants of 1.84 kg, while the application of R led to the highest weight of 10 plants at 2.50 kg. The application of Non-AZ exhibited a mean weight of 10 plants of 1.95 kg, while the application of AZ led to the highest weight of 10 plants at 2.20 kg. In 2022/2023, the R treatment again resulted in the largest weight of 10 plants, with a mean weight of 2.52 kg, while the control group exhibited the smallest weight at 1.89 kg. Overall, the R treatment had the most substantial positive impact on the weight of 10 plants, followed by RV and V. The application of Non-AZ exhibited a mean weight of 10 plants of 2.01 kg, while the application of AZ led to the highest average weight of 2.27 kg. The results of the interaction between the two factors showed that the lowest values were under the control application without organic fertilizer (C + Non-AZ) during both seasons (1.66 kg and 1.76 kg, respectively), while the highest values were under the application of rabbit manure with bio-fertilizer (R + AZ) during both seasons (2.54 kg and 2.61 kg, respectively).

**Table 5:** The integrated effect of Rabbit manure, Vermicompost treatments and their combinations with and without biofertilizer on yield variables of onion plants after two successive seasons (2021/2022–2022/2023)

Season	Treatment	Weight of 10 plant (kg)		Mean (A)	Yield (ton ha^−1^)		Mean (A)
		Non-AZ	AZ		Non-AZ	AZ	
2021/2022	C	**1** **.66 ± 0.07d**	2.02 ± 0.08c	1.84c	**41.64 ± 1.85d**	50.87 ± 1.99c	46.25c
	R	2.46 ± 0.10a	2.54 ± 0.10a	2.50a	61.94 ± 2.44a	63.86 ± 2.55a	62.90a
	V	1.76 ± 0.08d	2.03 ± 0.09c	1.89c	44.22 ± 1.89d	51.03 ± 2.27c	47.63c
	RV	1.92 ± 0.09c	2.22 ± 0.09b	2.07b	48.25 ± 2.27c	55.84 ± 2.19b	52.04b
	Mean (B)	1.95b	2.20a		49.01b	55.40a	
LSD (*p* ≤ 0.05)		**A = 0.01, B = 0.08, AB = 0.16**			**A = 2.84, B = 2.01, AB = 4.02**		
2022/2023	C	**1.76 ± 0.06d**	2.04 ± 0.08c	1.89d	**44.20 ± 1.63d**	51.30 ± 1.90c	47.75d
	R	2.43 ± 0.08b	2.61 ± 0.07a	2.52a	61.01 ± 1.91b	65.64 ± 1.76a	63.33a
	V	1.86 ± 0.09d	2.14 ± 0.07c	1.99c	46.69 ± 2.14d	53.76 ± 1.85c	50.23c
	RV	2.01 ± 0.09c	2.31 ± 0.14b	2.16b	50.46 ± 2.14c	58.18 ± 3.40b	54.32b
	Mean (B)	2.01b	2.27a		50.59b	57.22a	
LSD (*p* ≤ 0.05)		**A = 0.09, B = 0.07, AB = 0.01**			**A = 2.38, B = 1.68, AB = 3.36**		

The values shown in the table are the means of three replicates. Means followed by the same letters are non-significantly different (*p* ≤ *0.05*). Where: C = (100% mineral fertilizer at the full recommended rate); R = (100% Rabbit Manure); V = (100% Vermicompost); RV = (50% Rabbit Manure + 50% Vermicompost); Non-AZ = without *Azotobacter chroococcum;* AZ = with *Azotobacter chroococcum*

The results presented in Table 5 indicate that the yield (ton ha^−1^) was significantly affected by the various treatments across both growing seasons. In 2021/2022, the C exhibited a mean yield of 46.25 ton ha^−1^, while the application of R led to the highest yield at 62.90 ton ha^−1^. The application of Non-AZ exhibited a mean yield of 49.01 ton ha^−1^, while the application of AZ led to the highest yield at 55.40 ton ha^−1^. In 2022/2023, the R treatment again resulted in the largest yield, with a mean yield of 63.33 ton ha^−1^, while the control group exhibited the smallest yield at 47.75 ton ha^−1^. The application of Non-AZ exhibited a mean yield of 50.59 ton ha^−1^, while the application of AZ led to the highest average yield of 57.22 ton ha^−1^. The results of the interaction between the two factors showed that the lowest yield was under the control application without organic fertilizer (C + Non-AZ) during the two seasons (41.64 ton ha^−1^ and 44.20 ton ha^−1^, respectively), while the highest yield was under the application of rabbit manure with bio-fertilizer (R + AZ) during the two seasons (63.86 ton ha^−1^ and 65.64 ton ha^−1^, respectively).

### Impact of treatments on some soil properties

The effects of organic fertilizers like V and R alone or joint with bio-fertilizer AZ had a significant effect (*p* ≤ *0.05*) on some soil properties such as soil reaction (pH), electrical conductivity (EC), and organic matter (OM) were observed in both seasons (Table 6). However, the application of (V and R) played a crucial role in determining these parameters. Herewith are the findings, pH and EC significantly reduced value from 8.33 to 8.11 and 8.13 to V and R respectively, in the second season under bio-fertilizer, while EC reduced by 6.89 and 12.27% respectively, in the second season compared to the control. In contrast, R and `RV induced a significant increase in OM by 9.61% and 32.05%, in the first season, in the same regard, increments by 38.48 and 41.48%, respectively, in the second season compared to control.

**Table 6:** The integrated effect of Rabbit manure, Vermicompost treatments and their combinations with and without biofertilizer on some soil properties variables of onion plants after two successive seasons (2021/2022–2022/2023)

Season	Treatment	pH (1:2.5)		Mean (A)	ECdsm^−1^ (1:1)		Mean (A)	OM (gkg^−1^)		Mean (A)
		Non-AZ	AZ		Non-AZ	AZ		Non-AZ	AZ	
2021/2022	C	8.33 ± 0.06a	8.26 ± 0.03a	8.29A	1.07 ± 0.12a	1.06 ± 0.04a	1.06A	16.95 ± 0.47c	17.85 ± 0.52c	17.41C
	R	8.25 ± 0.11bcd	8.08 ± 0.11 cd	8.11B	0.87 ± 0.07a	1.00 ± 0.46a	0.97A	21.01 ± 1.39b	20.97 ± 1.25a	22.52A
	V	8.12 ± 0.03ab	8.10 ± 0.13 cd	8.16B	0.87 ± 0.11a	1.07 ± 0.09a	0.94A	21.37 ± 1.71b	23.66 ± 1.17b	20.19B
	RV	8.21 ± 0.03abc	8.02 ± 0.09d	8.11B	1.00 ± 0.05a	1.11 ± 0.10a	1.05A	20.38 ± 1.47b	22.38 ± 0.62ab	21.38AB
	Mean (B)	8.23A	8.12B		0.95A	1.06A		19.93B	21.22A	
LSD (*p* ≤ 0.05)		A = 0.10, B = 0.07, AB = 0.15			A = 0.23, B = 0.16, AB = 0.32			A = 1.44, B = 1.02, AB = 2.03		
2022/2023	C	8.40 ± 0.06a	8.33 ± 0.03ab	8.36A	1.10 ± 0.10ab	1.06 ± 0.04ab	1.08A	17.75 ± 0.42c	18.73 ± 0.52c	18.24B
	R	8.28 ± 0.11cde	8.11 ± 0.11cde	8.14B	0.91 ± 0.05c	1.02 ± 0.15bc	0.93B	23.08 ± 1.23ab	23.04 ± 0.88a	24.13A
	V	8.15 ± 0.03abc	8.13 ± 0.13de	8.19B	0.89 ± 0.08c	0.96 ± 0.09abc	0.97B	23.67 ± 1.71ab	24.58 ± 1.17ab	23.06A
	RV	8.23 ± 0.02bcd	8.05 ± 0.09e	8.14B	1.03 ± 0.05abc	1.13 ± 0.11a	1.08A	22.38 ± 1.47b	25.11 ± 1.49a	23.75A
	Mean (B)	8.26A	8.16B		0.98A	1.04A		21.72B	22.86A	
LSD (*p* ≤ 0.05)		A = 0.11, B = 0.07, AB = 0.14			A = 0.10, B = 0.07, AB = 0.15			A = 1.48, B = 1.05, AB = 2.09		

The values shown in the table are the means of three replicates. Means followed by the same letters are non-significantly different (*p* ≤ *0.05*). Where: C = (100% mineral fertilizer at the full recommended rate); R = (100% Rabbit Manure); V = (100% Vermicompost); RV = (50% Rabbit Manure + 50% Vermicompost); Non-AZ = without *Azotobacter chroococcum;* AZ = with *Azotobacter chroococcum*

The responses of onion plants amended of vermicompost and rabbit manure on soil nutrient availability and their uptake were with AZ or without bio-fertilizer Non-AZ (Table 7). The results indicated highly significant effects (*p* < 0.05) for available nutrients N, P, and K by 73.03, 26.18 and 29.41%, moreover, RV increased by 103.52, 30.33 and 4.24%respectively over the control in response to R with bio-fertilizer AZ in the second season.

**Table 7:** The integrated effect of Rabbit manure, Vermicompost treatments and their combinations with and without biofertilizer on uptake variables of onion plants (N, P and K) after two successive seasons (2021/2022–2022/2023)

Season	Treatment	N (kg ha^−1^)		Mean (A	P(kg ha^−1^)		Mean (A)	K(kg ha^−1^)		Mean (A)
		Non-AZ	AZ		Non-AZ	AZ		Non-AZ	AZ	
2021/2022	C	61.53 ± 1.43d	81.07 ± 6.59b	71.3c	29.86 ± 1.74f	44.81 ± 5.04 cd	37.34c	142.904.60 ± f	179.50 ± 2.36d	161.20d
	R	72.38 ± 2.06d	81.52 ± 4.05b	76.95b	31.94 ± 2.48e	49.06 ± 3.63b	40.50b	165.01 ± 5.69d	192.00 ± 7.31b	178.51b
	V	65.21 ± 2.21c	84.53 ± 1.55b	74.87b	36.26 ± 3.54ef	51.56 ± 1.17bc	43.91bc	175.34 ± 4.88e	200.86 ± 3.22c	188.10c
	RV	81.97 ± 3.46b	102.94 ± 5.53a	92.45a	43.22 ± 2.17d	69.17 ± 3.60a	56.20a	180.35 ± 0.64d	224.73 ± 4.53a	202.54a
	Mean (B)	70.27b	87.18a		35.32b	53.65a		165.90b	199.27a	
LSD (*p* ≤ 0.05)		A = 3.44, B = 2.43, AB = 4.86			A = 3.58, B = 2.54, AB = 5.07			A = 5.86, B = 4.14, AB = 8.28		
2022/2023	C	66.43 ± 4.80d	82.97 ± 6.59b	74.70b	30.32 ± 1.74f	49.52 ± 1.17 cd	39.92c	146.78 ± 4.60f	196.99 ± 3.22c	171.89d
	R	73.17 ± 3.57 cd	82.31 ± 4.52b	77.74b	32.94 ± 2.47e	52.54 ± 5.10b	46.57b	169.99 ± 5.69d	200.47 ± 7.31b	193.64b
	V	68.97 ± 2.21c	85.40 ± 1.55b	77.18b	37.24 ± 3.54ef	55.91 ± 3.63bc	42.74bc	179.22 ± 4.88e	206.85 ± 5.25bc	185.24c
	RV	85.53 ± 4.05b	103.94 ± 5.53a	94.74a	45.81 ± 5.04d	70.22 ± 3.60a	58.02a	185.49 ± 2.36d	230.85 ± 4.53a	208.17a
	Mean (B)	73.52b	88.66a		36.58b	56.05a		170.37b	208.79a	
LSD (*p* ≤ 0.05)		A = 4.05, B = 2.86, AB = 5.72			A = 4.01, B = 2.84, AB = 5.67			A = 6.33, B = 4.47, AB = 8.95		

The values shown in the table are the means of three replicates. Means followed by the same letters are non-significantly different (*p* ≤ *0.05*). Where: C = (100% mineral fertilizer at the full recommended rate); R = (100% Rabbit Manure); V = (100% Vermicompost); RV = (50% Rabbit Manure + 50% Vermicompost); Non-AZ = without *Azotobacter chroococcum;* AZ = with *Azotobacter chroococcum*

Amending the soil with vermicompost and rabbit manure with or without bio-fertilizer also significantly affected the relevant nutrient uptake (Table 8). Under the influence of RM100 treatment with bio-fertilizer increased N, P, and K uptake by 28.56, 84.40, and 40.92%, respectively, over control in the second season, while the highest value of treatment [RV + AZ] increased by 56.46, 131.60and 57.27% in the second season compared to control.

**Table 8:** The integrated effect of Rabbit manure, Vermicompost treatments and their combinations with and without biofertilizer on availability nutrients in soil of onion plants (N, P and K) after two successive seasons (2021/2022–2022/2023)

Season	Treatment	N (mg kg^−1^)		Mean (A)	P(mg kg^−1^)		Mean (A)	K(mg kg^−1^)		Mean (A)
		Non-AZ	AZ		Non-AZ	AZ		Non-AZ	AZ	
2021/2022	C	43.18 ± 0.94f	62.66 ± 1.06 cd	52.92c	7.47 ± 0.49c	8.54 ± 0.48abc	8.01b	168.15 ± 4.19e	196.28 ± 4.04c	182.21c
	R	54.01 ± 1.78e	65.97 ± 2.95b	63.79b	7.95 ± 0.69bc	8.70 ± 0.76ab	8.51ab	187.73 ± 5.82 cd	211.69 ± 2.68b	205.05b
	V	54.81 ± 3.16e	72.79 ± 0.99c	59.99b	8.18 ± 0.89bc	8.82 ± 0.59ab	8.33b	192.62 ± 7.04d	217.47 ± 3.27b	199.71b
	RV	58.45 ± 2.97de	86.73 ± 7.14a	72.58a	8.77 ± 0.31ab	9.68 ± 0.71a	9.23a	200.73 ± 3.05c	241.10 ± 4.66a	220.92a
	Mean (B)	52.61b	72.04a		8.09b	8.94a		187.31b	216.63a	
LSD (*p* ≤ 0.05)		A = 4.04, B = 2.85, AB = 5.71			A = 0.84, B = 0.59, AB = 1.19			A = 5.78, B = 4.09, AB = 8.18		
2022/2023	C	46.87 ± 0.92e	66.90 ± 1.62d	56.88d	7.78 ± 0.43d	8.86 ± 0.47bcd	8.32c	175.59 ± 2.67e	203.98 ± 3.58 cd	189.79c
	R	62.80 ± 1.78d	74.22 ± 1.47b	71.9b5	8.41 ± 0.53dc	9.03 ± 0.32ab	9.39ab	199.60 ± 5.46d	222.29 ± 2.24b	214.47b
	V	62.79 ± 3.16d	81.10 ± 0.84c	68.51c	8.97 ± 0.89 cd	9.82 ± 0.51abc	8.72bc	201.71 ± 4.05d	227.23 ± 2.01b	210.95b
	RV	67.10 ± 2.44d	95.39 ± 5.68a	81.24a	9.54 ± 0.32ab	10.14 ± 0.96a	9.84a	208.31 ± 2.12c	248.01 ± 2.02a	228.16a
	Mean (B)	59.89b	79.40a		8.67b	9.46a		196.30b	225.38a	
LSD (*p* ≤ 0.05)		A = 3.33, B = 2.35, AB = 4.71			A = 0.79, B = 0.56, AB = 1.12			A = 4.08, B = 2.88, AB = 5.77		

The values shown in the table are the means of three replicates. Means followed by the same letters are non-significantly different (*p* ≤ *0.05*). Where: C = (100% mineral fertilizer at the full recommended rate); R = (100% Rabbit Manure); V = (100% Vermicompost); RV = (50% Rabbit Manure + 50% Vermicompost); Non-AZ = without *Azotobacter chroococcum;* AZ = with *Azotobacter chroococcum*

### Impact of treatments on chemical metabolites in onion bulb

**Fig. 2:**
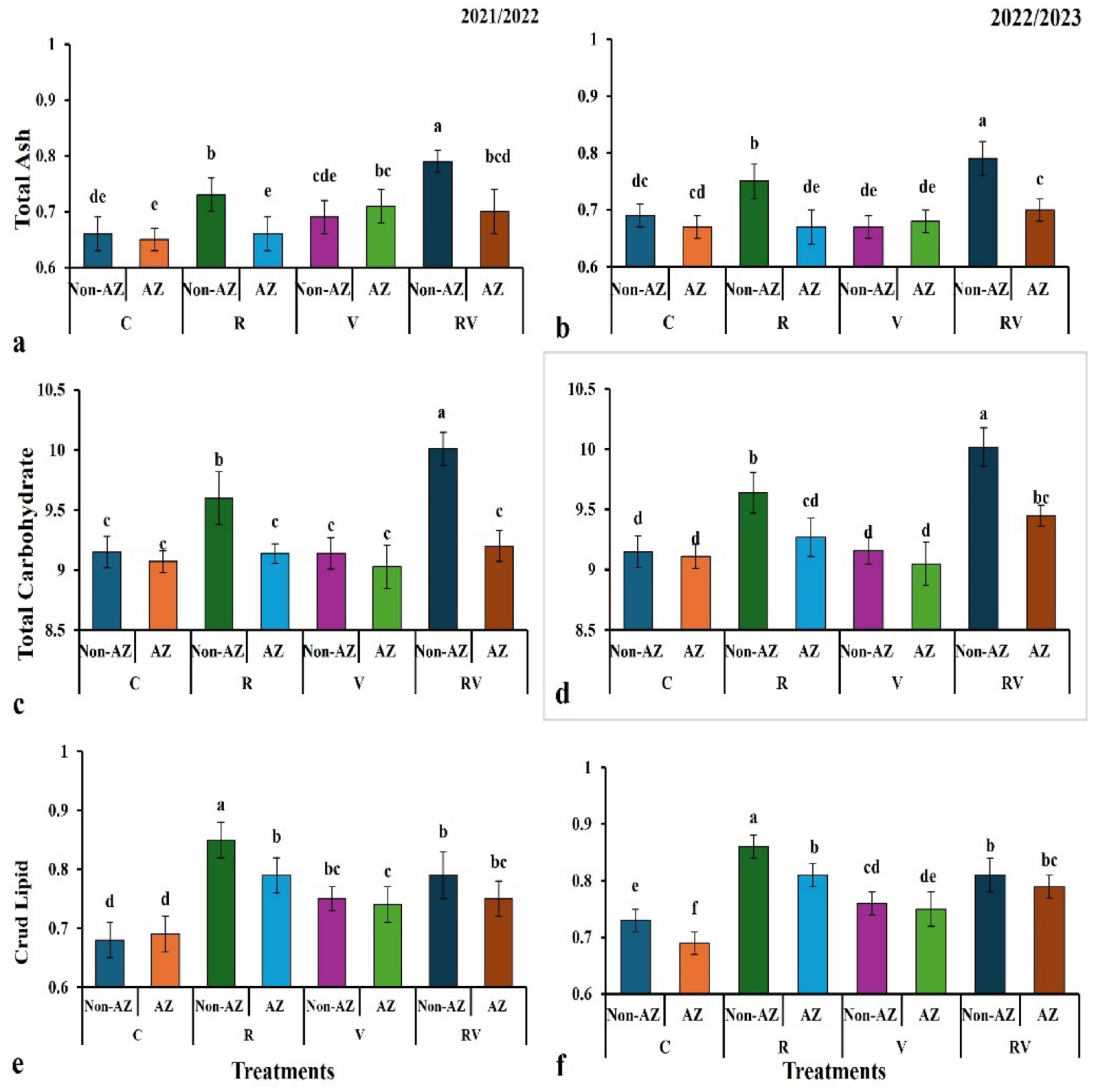
The integrated effect of organic fertilizers and bio-fertilizer on chemical compositions [total ash content, total carbohydrate content, and crud lipid content of onion plants after two successive seasons. The values shown are means three replicates. Means followed by the same letters are non-significantly different (*p* ≤ *0.05*). Where: C = (100% mineral fertilizer at the full recommended rate); R = (100% Rabbit Manure); V = (100% Vermicompost); RV = (50% Rabbit Manure + 50% Vermicompost); Non-AZ = without *Azotobacter chroococcum*; AZ = with *Azotobacter chroococcum*

The figure presents the total ash content for different treatments across two years (2021/2022 in Fig. 2a and 2022/2023 in Fig. 2b), showing the percentage increase in ash content compared to the C. In both years, treatments with Azotobacter (AZ) exhibit a higher total ash content than the control, with the R (AZ) group showing the largest percentage increase—21.54% in 2021/2022 and 17.91% in 2022/2023. The V (AZ) group shows a 12.31% increase in 2021/2022, but only a modest 1.49% increase in 2022/2023. The RV (AZ) group shows a 9.23% increase in 2021/2022 and 11.94% in 2022/2023. These results suggest that *Azotobacter* inoculation significantly enhances ash content, particularly in the R and RV treatments, with the effect being somewhat more pronounced in the first year.

The figure presents the total carbohydrate content for different treatments across two years (2021/2022 in Fig. 2c and 2022/2023 in Fig. 2d), showing the percentage increase in carbohydrate content compared to the C. In both years, the *Azotobacter* (AZ) treatments showed a slight increase in total carbohydrates compared to the control. The R (AZ) group exhibits the largest increase, with a 10.01% value in 2021/2022 and 10.02% in 2022/2023, while RV (AZ) shows a modest increase in both years (9.76% in 2021/2022 and 9.64% in 2022/2023). Other treatments like V (AZ) and C (AZ) show minimal variations across both years, with carbohydrate content remaining around 9.14–9.27. Overall, *Azotobacter* inoculation led to an increase in carbohydrates, especially in the R and RV treatments, with a relatively stable effect observed across both years.

The figure shows the percentage increase in crude lipid content for different treatments across two years (2021/2022 in Fig. 2e and 2022/2023 in Fig. 2f), comparing *Azotobacter* inoculated (AZ) and non-inoculated treatments (Non-AZ). In both years, the R (AZ) treatment demonstrates the highest percentage increase, with an increase of 25.0% in 2021/2022 (from 0.68 in the control to 0.85) and 24.6% in 2022/2023 (from 0.69 in the control to 0.86). The V (AZ) and RV (AZ) treatments show more moderate increases in crude lipids, ranging from 9.6 to 17.4%, with RV (AZ) showing a consistent increase of 14.5% in both years. The control groups (C) have the lowest lipid content, and *Azotobacter* inoculation generally leads to an increase, particularly in the R treatment, highlighting its potential role in boosting lipid accumulation.

**Fig. 3:**
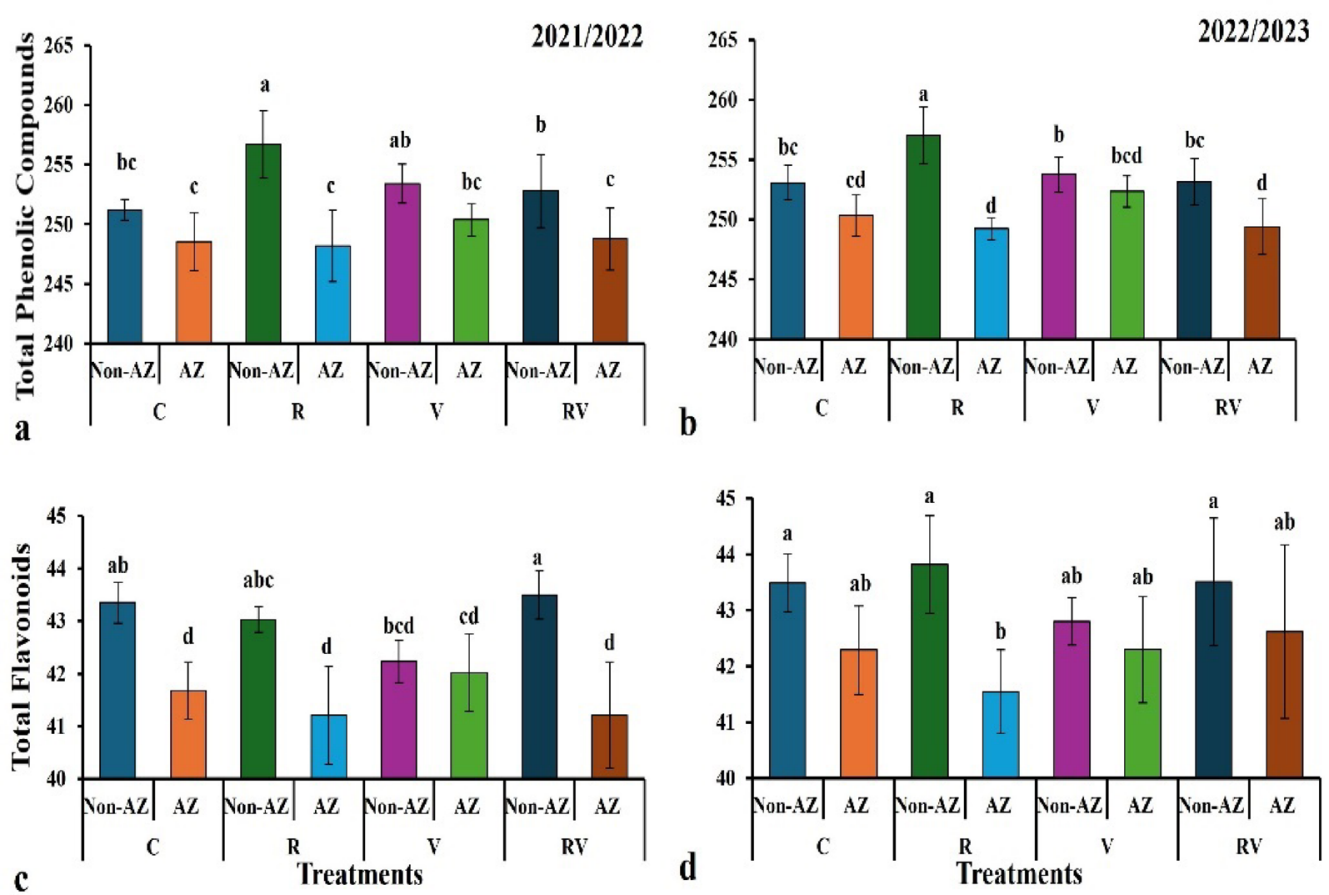
The integrated effect of organic fertilizers and bio-fertilizer on chemical compositions [total phenolic compounds and total flavonoids of onion plants after two successive seasons. The values shown are means three replicates. Means followed by the same letters are non-significantly different (*p* ≤ *0.05*). Where: C = (100% mineral fertilizer at the full recommended rate); R = (100% Rabbit Manure); V = (100% Vermicompost); RV = (50% Rabbit Manure + 50% Vermicompost); Non-AZ = without *Azotobacter chroococcum;* AZ = with *Azotobacter chroococcum*

The figure shows the percentage increase in total phenolic compounds for different treatments across two years (2021/2022 in Fig. 3a and 2022/2023 in Fig. 3b), comparing *Azotobacter* inoculation (AZ) with non-inoculated treatments (Non-AZ). In both years, the R (AZ) treatment demonstrates the highest percentage increase in total phenolic compounds, with an increase of 3.3% in 2021/2022 (from 248.52 in the control to 256.69) and 3.3% in 2022/2023 (from 253.07 in the control to 257.03). The RV (AZ) treatment shows a moderate increase of 1.7% in 2021/2022 (from 248.76 in the control to 252.78) and 1.6% in 2022/2023 (from 249.42 in the control to 253.14). Other treatments like V (AZ) and C (AZ) show minimal variations, with percentage increases ranging from 0.2 to 2.5%. These results indicate that *Azotobacter* inoculation increases phenolic compound content, particularly in the R treatment, with relatively stable effects observed across the two years.

The figure shows the percentage increase in total flavonoid content for different treatments across two years (2021/2022 in Fig. 3c and 2022/2023 in Fig. 3d), comparing *Azotobacter* inoculation (AZ) with non-inoculated treatments (Non-AZ). In both years, the R (AZ) treatment demonstrates the highest percentage increase in flavonoid content, with a 5.97% increase in 2021/2022 (from 41.21 in the control to 43.5) and 5.75% in 2022/2023 (from 41.55 in the control to 43.82). The RV (AZ) treatment shows a moderate increase of 5.18% in 2021/2022 (from 41.68 in the control to 43.35) and 4.52% in 2022/2023 (from 42.8 in the control to 43.49). Other treatments, like V (AZ) and C (AZ), show smaller variations, with increases ranging from 0.51 to 2.46%. These results indicate that *Azotobacter* inoculation increases flavonoid content, particularly in the R and RV treatments, with a consistent trend observed across both years.

## Discussion

Rabbit manure (R) consistently resulted in the highest mean plant height across both seasons, significantly outperforming the C and other treatments. This can be attributed to the high nutrient content of rabbit manure, particularly nitrogen, which is essential for vegetative growth (Waruwu et al. 2021; Ano and Ekefan 2023; Mmbaga et al. 2024; Wysokinski and Kożuchowska 2024). The slow-release nature of organic nutrients in rabbit manure ensures sustained availability throughout the growing season (Gideon et al. 2023), promoting continuous plant development (Li et al. 2022). These findings align with studies by Ariani et al. (2025), who reported similar improvements in plant height with organic amendments rich in nitrogen. The combination of rabbit manure with *Azotobacter* (R + AZ) yielded the highest plant heights, suggesting a synergistic effect between organic matter and beneficial microorganisms (Sayed et al. 2024). The *Azotobacter* is likely to enhance nutrient uptake and root development, further boosting plant growth (Minuț et al. 2022). In contrast, the lowest values were observed under the control treatment without *Azotobacter* (C + Non-AZ), highlighting the importance of both organic amendments and microbial activity in optimizing plant height.

Rabbit manure (R) significantly increased the weight of 10 onion plants, with the highest values recorded under the R + AZ treatment. This demonstrates the economic and agronomic potential of integrating rabbit manure and *Azotobacter* to enhance onion productivity. In contrast, the lowest yields were observed in the control without bio-fertilizer (C + Non-AZ), highlighting the critical role of these amendments in sustainable agriculture. The yield improvements can be attributed to enhanced nutrient availability and improved root development promoted by both rabbit manure and *Azotobacter* (Minuț et al. 2022). Consistently across both seasons, the R treatment produced superior yields, especially when combined with bio-fertilizer. These findings are consistent with Syamsi et al. (2021) who reported that incorporating 25–50% rabbit manure improved plant growth parameters such as leaf length, number of leaves, bulb diameter, and bulb weight, while also enhancing soil properties including pH, nitrogen, phosphorus, and cation exchange capacity, and reducing potassium content in ultisol soil.

The results of this study clearly demonstrate that integrating organic fertilizers, particularly rabbit manure and vermicompost, with the bio-fertilizer *Azotobacter* positively influenced soil health and onion productivity. The observed improvements in soil pH, electrical conductivity (EC), and organic matter (OM) content highlight the capacity of organic amendments to enhance key soil properties critical for sustainable crop production (Syamsi et al. 2021). Rabbit manure and vermicompost not only supplied essential macro- and micronutrients but also improved soil structure and water-holding capacity, creating a more favorable environment for plant growth (Alejo Jr and Nicolas 2021; Hussain and Abbasi 2018). The reduction in EC under the R + AZ treatment suggests effective salinity regulation, possibly due to increased cation exchange capacity (Al-Sayed et al. 2024), while the slight decrease in soil pH may have facilitated better nutrient availability (Barrow and Hartemink 2023; Qi 2024). Moreover, the significant rise in OM under integrated treatments points to improved microbial activity and nutrient cycling, which are essential for maintaining long-term soil fertility (Jiao et al. 2021; Shu et al. 2022). These findings reinforce the potential of integrated nutrient management approaches in arid and semi-arid environments, where maintaining soil quality is crucial for sustainable agricultural productivity.

In terms of nutrient availability, the observed increases in nitrogen (N), phosphorus (P), and potassium (K) resulting from the combined application of *Azotobacter* and organic fertilizers are in line with previous findings. *Azotobacter* is well-documented for enhancing nitrogen availability through biological nitrogen fixation, contributing to improved soil fertility (Aasfar et al. 2021, Martin del Campo et al. 2022). The synergistic interaction between *Azotobacter* and organic inputs such as rabbit manure and vermicompost likely facilitated greater nutrient mineralization and mobilization, leading to enhanced nutrient uptake by onion plants (Roy et al. 2022). This increased nutrient acquisition reflects not only a higher availability of essential elements in the soil but also improved root absorption efficiency, which collectively contributed to superior plant growth and yield performance (Ghadimi et al. 2021). These results emphasize the value of integrated nutrient management in achieving both agronomic and environmental sustainability.

The application of *Azotobacter*, particularly in combination with R and with RV treatment, significantly enhanced the chemical composition of onion bulbs. Notably, increases in ash content, carbohydrates, lipids, phenolic compounds, and flavonoids underscore the pivotal role of *Azotobacter* in improving both the nutritional quality and bioactive profile of onions. Elevated ash content indicates greater mineral accumulation, likely due to *Azotobacter*’s ability to facilitate nutrient uptake—especially nitrogen, a critical element for plant metabolism and growth (Darakeh et al. 2022; Barogh et al. 2023). The rise in carbohydrate levels further suggests improved photosynthetic efficiency and carbon assimilation, providing essential energy reserves that support both yield and quality attributes (Chawla and Sharma 2024). These findings align with studies by Nida et al. (2024), who reported that the application of *Azotobacter* (*P1* and *P2*) also enhanced the quantum yield of electron transport (jEo), as well as the efficiencies of the light reaction (φPo/(1 − φPo)) and biochemical reaction (ψo/(1 − ψo)) in sub-optimal conditions in *Zea mays*. Additionally, the increase in lipid content implies a stimulation of lipid biosynthesis pathways, contributing to enhanced energy storage and metabolic activity (Zhou et al. 2023). In addition to the benefits of *Azotobacter*, the use of organic fertilizers, like rabbit manure and vermicompost, further boosted the synthesis of secondary metabolites such as phenolic compounds and flavonoids. These compounds are not only vital for the plant’s defense mechanisms but also offer antioxidant properties that can enhance the nutritional value of the onions (Bibi et al. 2022; Zhao et al. 2021).

## Conclusion

The results of this study demonstrate that the application of rabbit manure and *Azotobacter* bio-fertilizer significantly enhances the growth and yield of onion plants. Rabbit manure consistently outperformed other treatments, leading to the highest increases in plant height, leaf number, bulb diameter, and bulb weight. The combination of rabbit manure with *Azotobacter* (AZ) resulted in the most significant improvements in all morphological parameters, demonstrating a strong synergistic effect between organic fertilization and bio-fertilization. Moreover, the study highlights the positive impact of organic fertilization on soil properties, including increased organic matter content, reduced pH, and improved electrical conductivity, contributing to better soil health and nutrient availability. Additionally, the incorporation of *Azotobacter* significantly increased the chemical metabolites in onion bulbs, including phenolic compounds, flavonoids, and lipids, which are beneficial for both plant health and human nutrition. Plans should focus on conducting field trials over multiple years to evaluate the long-term effects of these treatments on onion yield, soil health, and metabolic profiles. Additionally, exploring the impact of *Azotobacter* in combination with different organic fertilizers on other crops would be valuable in extending these findings to broader agricultural systems. Further research on optimizing the application rates and timing of bio-fertilizers in various soil types would help refine sustainable farming practices and maximize benefits for both crop production and environmental health.

## Data Availability

No datasets were generated or analysed during the current study.
